# Regulation of Mitochondrial Dynamics in Parkinson’s Disease—Is 2-Methoxyestradiol a Missing Piece?

**DOI:** 10.3390/antiox10020248

**Published:** 2021-02-06

**Authors:** Paulina Bastian, Jaroslaw Dulski, Anna Roszmann, Dagmara Jacewicz, Alicja Kuban-Jankowska, Jaroslaw Slawek, Michal Wozniak, Magdalena Gorska-Ponikowska

**Affiliations:** 1 Department of Medical Chemistry, Medical University of Gdansk, Debinki 1, 80-211 Gdansk, Poland; paulina.przychodzen@gumed.edu.pl (P.B.); alicja.kuban-jankowska@gumed.edu.pl (A.K.-J.); mwozniak@gumed.edu.pl (M.W.); 2 Department of Neurological-Psychiatric Nursing, Medical University of Gdansk, 80-211 Gdansk, Poland; jaroslaw.dulski@gumed.edu.pl (J.D.); annaroszmann@gmail.com (A.R.); jaroslaw.slawek@gumed.edu.pl (J.S.); 3 Neurology & Stroke Dpt. St. Adalbert Hospital, “Copernicus” Ltd., 80-462 Gdansk, Poland; 4 Faculty of Chemistry, University of Gdansk, Wita Stwosza 63, 80-308 Gdansk, Poland; dagmara.jacewicz@ug.edu.pl; 5 Euro-Mediterranean Institute of Science and Technology, 90139 Palermo, Italy; 6 Department of Biophysics, Institute of Biomaterials and Biomolecular Systems, University of Stuttgart, 70174 Stuttgart, Germany

**Keywords:** 2-methoxyestradiol, oxidative stress, nitric oxide, Parkinson’s disease, mitochondria, cancer

## Abstract

Mitochondria, as “power house of the cell”, are crucial players in cell pathophysiology. Beyond adenosine triphosphate (ATP) production, they take part in a generation of reactive oxygen species (ROS), regulation of cell signaling and cell death. Dysregulation of mitochondrial dynamics may lead to cancers and neurodegeneration; however, the fusion/fission cycle allows mitochondria to adapt to metabolic needs of the cell. There are multiple data suggesting that disturbed mitochondrial homeostasis can lead to Parkinson’s disease (PD) development. 2-methoxyestradiol (2-ME), metabolite of 17β-estradiol (E2) and potential anticancer agent, was demonstrated to inhibit cell growth of hippocampal HT22 cells by means of nitric oxide synthase (NOS) production and oxidative stress at both pharmacologically and also physiologically relevant concentrations. Moreover, 2-ME was suggested to inhibit mitochondrial biogenesis and to be a dynamic regulator. This review is a comprehensive discussion, from both scientific and clinical point of view, about the influence of 2-ME on mitochondria and its plausible role as a modulator of neuron survival.

## 1. Parkinson’s Disease

Parkinson’s disease (PD) is the second most frequently diagnosed neurodegenerative disorder after Alzheimer’s disease (AD) with its prevalence being approximated at 0.3% of the total population and about 1% in group people over 60 years old [1]. In recent years, several genetic mutations have been identified to be associated with PD, however, the vast majority of PD cases (about 90%) occur in a sporadic manner [1]. Irrespective of its underlying cause (hereditary versus sporadic), PD is identified by preferential loss of dopaminergic neurons (DA) in the substantia nigra pars compacta that project to the basal ganglia [2]. The disruption of the nigrostriatal pathway, which projects from the pars compacta of the substantia nigra to the striatum and is particularly involved in the control of motor activity and motivated behaviors, results in the poverty of movement that characterizes PD. The nigrostriatal pathway resides in the ventrolateral cell groups within the substantia nigra which have pacemaker-like properties that may be associated with frequent intracellular calcium transients [2]. Together with the deficiency in calcium buffering that is often present in these cells, these may lead to cellular stress and interruption of cellular homeostasis [2]. The ensuing disruption of nuclear membrane stability results in release of histones, which induce oligomers rich in protofibrils and mature fibrils, and other proaggregant nuclear factors that may provoke α-synuclein (α-syn) aggregation [2,3]. The intraneuronal inclusions mainly composed of α-syn aggregates, known as Lewy bodies, are the hallmark of PD [2]. The specific molecular basis fundamental to DA neuron degeneration remains still insufficiently studied, however the clinical and pathological aspects of PD have been extensively described [4].

Clinically, PD is diagnosed by the presence of motor symptoms that includes bradykinesia and at least one of the following: rest tremor, rigidity and postural instability. However, nonmotor symptoms are present in the majority of PD cases and are pivotal players in determining the patient’s quality of life [5,6]. Recent studies have demonstrated that among the wide spectrum of nonmotor symptoms, sleep disorders in particular impact the quality of life [6]. Sleep–wake disturbances are common and affect 75–80% of patients with PD [7]. The most commonly occurring sleep disturbances include insomnia, sleep fragmentation, sleep-related breathing disorders, restless legs/periodic leg movements, REM sleep behavior disorder (RBD), nocturnal hallucinations, decreased sleep deficiency, sleep attacks, and excessive daytime sleepiness [7]. Interestingly enough, sleep–wake problems were demonstrated to be independent of motor disturbances and thus they may have another, other than simply dopaminergic, origin [6]. Furthermore, recently more data emerged, suggesting nondopaminergic systems involvement in the pathogenesis of the sleep and wake disturbances in PD [6]. As PD probably commences in the nondopaminergic structures of the brain or peripheral nervous system, the nonmotor symptoms are of particular importance [5]. The relationship between sleep and neurodegeneration in PD is still not fully understood, however, most probably it is bidirectional in nature [7,8]. Sleep may influence neurodegeneration by disease-modifying mechanisms such as activation of inflammation, disturbed nocturnal brain oxygenation, impaired proteostasis and synaptic homeostasis, alterations in glymphatic clearance, and altered variation of specific neuronal networks that may increase further propagation of α-synucleinopathy in the brain. Conversely, sleep–wake disturbances may be a manifestation of neurodegeneration and reflect the degree of brain damage [8].

The diagnosis and treatment of PD should address both motor and nonmotor symptoms of the disease. Symptomatic pharmacological treatment is available in PD. Unfortunately, there is still no curative drug, and the cause of the disease is not known. There has been great progress in understanding the pathophysiology of PD, including genetic and biochemical causes. Many years of research conducted on a large scale improved the recognition of many mechanisms influencing the occurrence of the symptoms of the disease, unfortunately, this did not significantly affect the course and modification of the disease. The factors that could prevent the progression of the disease are still not known. Unfortunately, treatment is limited to symptomatic methods. Presently, there is a number of different treatments methods for PD and for different stages of the disease but broadly they are classified into dopaminergic, nondopaminergic treatments, surgical possibilities, and device aided therapies [9,10].

Currently there is no disease-modifying treatment for PD, but the available medications can provide significant symptomatic benefits for patients with PD [11]. As dopamine itself cannot cross the blood–brain barrier it is not used in the treatment of PD [11]. Levodopa (L-dihydroxyphenylalanine or L-DOPA), the direct metabolic precursor of dopamine, brings the greatest symptomatic relief [11,12]. In order to minimize peripheral conversion by DOPA decarboxylase it is combined with peripheral inhibitors of DOPA decarboxylase (benserazide or carbidopa) [11]. Dopamine agonists bind directly to the dopaminergic receptors in the brain and stimulate the activity of the dopamine system [11]. Dopamine agonists are used as the mainstay of treatment in patients younger than 60 years as they may have lower potential to cause dopaminergic motor complications, in particular dyskinesia [11,12]. Amantadine, initially registered as an antiviral drug, is a glutamate antagonist at the N-methyl-D-aspartate receptor (NMDAR) and additionally displays weak dopamine releasing effects [11,12]. Monoamine Oxidase B (MAO-B) inhibitors (rasagiline, selegeline and safinamide) prevent the enzyme from metabolizing dopamine and thus prolong its duration of action [11]. Catechol-O-methyl transferase inhibitors (entacapone, tolcapone and opicapone) reduce dopamine breakdown by another enzyme and are used mainly as adjunctive therapy to L-DOPA [11]. They offer little clinical benefit in terms of the nonmotor manifestations of PD. It is usual practice to delay the initiation of treatment until the patient’s symptoms become troubling, to reduce the impact of adverse effects [11].

Despite all treatment possibilities the unmet need in PD is the development of treatment that slows or even stops the neurodegenerative processes in the brain. Disease-modifying treatments have not been developed yet. 

Another important aspect is the diversity of symptoms in PD patients, despite one diagnosis, the course of the disease is very diverse in different individuals. Perhaps there is not a single disorder pattern for all patients, but rather there are specific subtypes depending on unknown factors related to the heterogeneity of Parkinson’s disease picture and genetic background. It would be desirable to understand the underlying pathological processes. 

Understanding the molecular mechanisms in PD may provide targets and refine treatment.

It is worth pointing out that the molecular pathways of neurodegeneration and carcinogenesis may overlap [13,14,15,16,17,18]. Table 1 represents shared pathogenetic factors for cancers and neurodegerative disorders like PD. The recent studies indicate involvement of several neurodegeneration-causing factors like α-syn, PARK2 (Parkin), AMP-activated protein kinase (AMPK), and PARK5 in cancer development as regulators of apoptosis induction [15]. Loss of Parkin function was found in the case of PD and cancers. In addition, it was established that Parkin takes part in initiation of tumor formation process and its mutations were present in lung, liver, intestine, and brain cancers [19,20,21,22,23,24,25]. PINK1 deficiency impairs the plasticity of stratium and hippocampus in PD, but its high expression was observed in lung cancer [26,27,28,29]. Moreover, a family of synucleins were proved to be crucial, not only in PD development, but also in cancer pathogenesis due to its regulation and tumor differentiation [30,31]. Furthermore, selectively nitrated α-syn was established to directly induce nitro-oxidative stress and promote neurodegenerative diseases [32]. Accumulation and aggregation of α-syn was found in many types of cancers including melanoma, brain, and glial tumors [33,34,35,36,37]. Mutations in leucine rich repeat kinase 2 (LRRK2) and the genes coding synuclein underly autosomal dominant hereditary PD, while mutations in DJ-1, PARKIN, PINK1, are present autosomal recessive variations. As these proteins affect mitochondrial functioning, patients with PD suffer from mitochondrial mass shortage and autophagy pathway deterioration [38]. Above-mentioned research point a potential role of mitochondrial impairment in PD’s pathogenesis. Interestingly, comparable abnormalities are often described in carcinogenesis [39,40]. Interestingly, epidemiologic studies show a decreased incidence of most cancer types in PD except melanoma, brain tumors, thyroid and breast cancers [40]. 

The purpose of neuronal cell death in PD is still uncertain, however, the potential involvement of mitochondria, endoplasmic reticulum, α-syn, or dopamine were exposed as they contribute to cellular oxidative stress [41].

## 2. Biomarkers of Oxidative Stress in Physiology and Pathophysiology of Nervous System

Reactive oxygen species (ROS) and reactive nitrogen species (RNS) are vital signaling molecules produced by the aerobic metabolism [45]. Oxidation-reduction (redox) reactions and post-translational modifications of proteins are ways of signals transduction by ROS and RNS [46,47]. The mammalian brain is a key producer of ROS and RNS and redox signaling is crucial in the physiology of the healthy brain [42,45]. Under pathological conditions, ROS and RNS can reach excessive levels, generating oxidative and nitrosative stresses, resulting in damage DNA, lipid, and proteins disturbing, nonspecifically, cell function [44]. Nitro-oxidative stress contributes to the pathophysiological mechanisms in neurodegenerative disorders including PD. The understanding of biochemical processes involved in the maintenance of redox homeostasis in the brain has provided wider knowledge of mechanisms of neuroprotection and neurodegeneration [42,43,44,45].

ROS are oxygen-derived species and include hydrogen peroxide (H_2_O_2_), hydroxyl radical (^•^OH), superoxide (O_2_^•−^), hydroperoxyl radical (HO_2_^•^), peroxyl radical (ROO^•^), and singlet oxygen (^1^O_2_) [45]. ROS are highly reactive and a rapid cascade of transitions from one species to another is observed. Notably, the O_2_^•−^ is unstable and immediately dismutates into H_2_O_2_ by superoxide dismutase (SOD). When the O_2_^•−^ reacts with nitric oxide (^•^NO), then peroxynitrite (ONOO^–^) is produced. ^1^O_2_ is formed by the reaction of hypochlorous acid (HOCl) with H_2_O_2_ [44].

Main sources of ROS are cellular respiration and metabolic processes [44]. Major formation of ROS occur in normal cellular metabolism as mitochondrial electron transport chain, β-oxidation of fatty acids, cytochrome P450-mediated reactions, and by the respiratory burst during immune defense [48]. Oxidative phosphorylation in respiratory chain generates mitochondrial ROS. Electrons derived from NADH or FADH directly react with oxygen, O_2_^•−^, precursor of most ROS, or other electron acceptors and form free radicals [44]. In the cell the main sources are NADPH oxidases (NOX) and mitochondria. O_2_^•−^ is rapidly converted to H_2_O_2_ by SOD, which in comparison to O_2_^•−^ is more stable and durable. Moreover, due to its accelerated mobility, O_2_^•−^ can cross membranes relatively easily. It is reduced to water by catalase, glutathione peroxidase (GPX) and peroxiredoxins [43]. Furthermore, iron, in the redox cycle as a ferrous ion, converts H_2_O_2_, in the Fenton reaction, to produce a hydroxyl radical (^•^OH)—the extremely reactive and short-lived molecule that causes toxic oxidative damage to DNA, proteins, and membrane lipids [42,49,50]. 

Due to high oxygen consumption, low antioxidant defense, and an abundance of oxidation-sensitive lipids, the brain is very susceptible to ROS-mediated damage. ROS play an essential role in neurological disease progression as their high concentrations lead to pathology development. On the other hand, low ROS concentrations are crucial for proper brain functioning [44].

H_2_O_2_ migrates from its site of generation and function as a signaling molecule or second messenger as it is comparatively less reactive than O_2_^•−^ [51]. By synthesis of transcription factors, inhibition of the ubiquitin E3 ligase complex, exposure or masked nuclear localization signals, and modulation of transcription factor affinity towards deoxyribonucleic acid (DNA), coactivators or repressors, H_2_O_2_ regulates gene expression. The transcription factors that are controlled by H_2_O_2_ include *Escherichia coli* OxyR, NF-κB, activator protein-1, hypoxia-inducible factor-1 [52].

H_2_O_2_ is a byproduct of aerobic respiration in aerobic cells, but is also formed in enzymatic reactions in mitochondria and peroxisomes [53]. Tissue injury and inflammation results in elevated production of H_2_O_2_, which during tissue damage is an essential feature for wound healing response [54,55]. The production of H_2_O_2_ after damage is mostly mediated by NOX, which is expressed mainly on the mitochondrial and endoplasmic reticulum membrane [56,57,58]. After activation by, for instance, mechanical injury, pathogen attack, and inflammatory cytokines, NADPH oxidase transform one oxygen molecule into O_2_^−^ which quickly turns into H_2_O_2_ under the activity of SOD [59].

Recently it was demonstrated that respiring mitochondria are the fundamental source of H_2_O_2_ formation for dynamic neuronal signaling [60]. To investigate the biological roles of endogenous H_2_O_2_ in cell mitosis, Guo et al. develop a near-infrared ratiometric fluorescent probe Cy-PFS for specifically imaging endogenous H_2_O_2_ in cells and in vivo. The study examined H_2_O_2_ signaling in cell mitosis through growth factor signaling in live hippocampal neurons cells. Furthermore, the close association of endogenous H_2_O_2_ level changes with the brain development was successfully demonstrated at various stages [61]. Tabner et al. demonstrated that H_2_O_2_ is produced from an aggregating β-amyloid or α-syn by a Fe (II)-dependent mechanism, and that this is subsequently converted to ^•^OH, by Fenton’s reaction. Consequently, one of the fundamental molecular mechanisms underlying the pathogenesis of cell death in AD and PD, and possibly other neurodegenerative or amyloid diseases, could be the direct production of H_2_O_2_ during formation of the abnormal protein aggregates [62].

## 3. Mitochondrial Antistress Protective Systems

In order to protect the cells from harmful ROS, mitochondria developed their specific antioxidant system for the scavenging and detoxification of the radicals produced [63]. In the process of neutralization of H_2_O_2_ ebzymes are involved such as Glutathione Peroxidase-1 (GPx-1) [64], phospholipid-hyperoxide glutation peroxidase (PhGPx) [65], and catalase (CAT) or peptides like glutathione (GSH) [66,67,68]. GPx-1 is a selenoprotein, found in the cytoplasm and matrix of mitochondria, that belongs to peroxidases that reduce peroxides using reducing properties of glutathione. GPx-1 and PhGPx are the two mitochondrial forms of GPx, with PhGPx found in the matrix and PHGPX rooted in the inner membrane [69]. GPx-1 is ubiquitously expressed in eukaryotic cells where it reduces hydrogen and lipid peroxides to alcohols [70]. GPx-1 overexpression, both in vivo and in vitro models, results in enhanced protection against oxidative stress [70,71]. GPx-1 deficiency is correlated with higher vulnerability to oxidant stress. Mice lacking GPx-1 suffer from endothelial dysfunction and abnormalities in vascular and cardiac structure [72,73]. Moreover, in cerebral models of ischemia-reperfusion, GPx-1-deficent mice were more prone to injury [74]. While overexpression of GPx-1 may be protective against oxidative stress generated in a various diseases, excess GPx-1 may disturb normal oxidative signaling due to extensive removal of endogenous intracellular H_2_O_2_ [64]. Moreover, in ischemic rats, Gpx-1 overexpression significantly reduced cytosolic translocation of cytochrome c and decreased apoptosis-related factors such as Bax and activated caspase 3 [75]. 

Another crucial enzyme for H_2_O_2_ destruction is CAT [76]. In human organisms it is present in every organ and the highest levels are observed in the liver, kidney and red blood cells [77]. Interestingly, decreased CAT activity in the substantia nigra and putamen of the parkinsonian brain were detected [78]. That proves that deficiency or malfunction of these enzymes result in the pathogenesis of many age-associated degenerative diseases, including PD [48,78]. Moreover, CAT deficit is related to many diseases including metabolic disorders such as type I and II diabetes [79,80], hypertension [81] and insulin resistance [82], but also neurological disorders like PD [78,83], AD [84,85], bipolar disorder [86,87], and schizophrenia [88]. What is more, modification of CAT expression has been described in cancer tissues [76,89], and its gene polymorphism was proven to be a reason of susceptibility to ovarian cancer [90]. Remarkably, there are multiple studies on usage of CAT in anticancer therapy [91,92,93]. 

SODs are a group of metalloenzymes, that constitute the front line of defense against ROS-mediated injury. They are responsible for the dismutation of O_2_^-^ into H_2_O_2_ [94]. Depending on the metal atom present in the active sites, SODs can be divided into four separate groups: Copper-Zinc-SOD (Cu, Zn-SOD) [95], Iron SOD (Fe-SOD) [96], Manganese SOD (Mn-SOD) [97], and Nickel SOD [98]. CuZnSOD, also known as SOD1, is a homodimer predominantly localized in the cytoplasm, but only slight quantities have been detected in the intermembrane space of mitochondria [99]. Human skin Cu, Zn-SOD is an enzyme that protects skin against ROS and is present in the cytoplasm of keratinocytes, where even 90% of cellular ROS is formed [95]. Fe-SOD is an antioxidant of bacterial origin present for instance in *E. coli* [96]. The mitochondrial MnSOD, named also SOD2, is considered as the chief ROS scavenging enzyme in the cell [97]. MnSOD is reduced in many types of cancers, including breast [100,101,102], pancreatic [103] and ovarian cancers [104,105], but it may act as either a tumor suppressor or a tumor promoter depending on cancer type. On the one hand, improved metastasis by MnSOD is a result of stimulation of its expression through H_2_O_2_-dependent mechanisms [106]. However, on the other hand, overexpression of MnSOD prevents from various hallmarks features of cancer, like augmented growth rate and invasiveness [107,108]. Within the SOD family, NiSOD is rather extraordinary, because nickel is the only metal unable to catalyze O_2_^•−^ dismutation, apparently due to a lack of an accessible one-electron redox process [98,109]. However, physiological function of NiSOD is still unknown.

## 4. Nitric Oxide as an Ignition Link of Apoptosis

Nitric oxide (NO) functions as a reversing neurotransmitter in synapses, by widening the blood vessels allowing the brain blood flow and playing many key roles in intracellular signaling in neurons from the regulation of the neuronal metabolism to the dendritic spine development [110]. 

^•^NO is synthetized from L-arginine, nicotinamide adenine dinucleotide phosphate (NADPH) and oxygen due to activity of the nitric oxide synthase family (NOS), using flavin adenin dinucleotide (FAD), flavin adenin mononucleotid (FMN), tetrahydrobiopterin (BH4) and calmodulin [111]. The family of NOS contains three isoforms: neuronal NOS (nNOS), inducible NOS (iNOS) and endothelial NOS (eNOS) [111]. nNOS, known also as NOS I, is constitutively present in central and peripheral neurons [112]. iNOS (NOS II) can be expressed in various cell types in response to lipopolysaccharide, cytokines, or other agents. It plays a key role in inflammatory diseases and septic shock generating large amounts of NO that have cytostatic effects [113]. eNOS (NOS III) is mainly identified in endothelial cells. It keeps blood vessels dilated, controls blood pressure, and has several other vasoprotective and antiatherosclerotic features [114]. 

The NO formed by NOS can affect abundant types of enzymes and proteins. The activation of soluble guanylyl cyclase and the generation of cyclic GMP is a key signal transducing pathway stimulated by NO [111]. The derivatives of nitric oxide, such as nitrogen dioxide (NO_2_) and ONOO^-^ cause protein and lipid peroxidation and DNA damage resulting in cell death. Besides, due to control of the levels of proteins significant for cell survival such as BAX and Bcl-2, they are vital players within the pro- and antiapoptotic molecular pathways [115,116]. Additionally, NO performs post-translational modifications in proteins by the S-nitrosylation of the thiol group of cysteine residue in peptide or proteins, which is a physiological mechanism to regulate protein function [117]. Protein S-nitrosylation is an irreversible process that also results in the accumulation of modified proteins that contribute to the emergence and development of neurodegenerative disorders such as AD or PD [110].

## 5. 2-Methoxyestradiol (2-ME) a Physiological Compound and an Anticancer Agent

2-methoxyestradiol (2-ME) is a physiological compound, a metabolite of 17β-estradiol (E2), which belongs to estrogens, female sex hormones [118]. Additionally, 2- ME is a presumably effective anticancer agent [119]. Under the trade name Panzem, it has been evaluated in advanced stages of clinical trials for the treatment of various types of cancers, including colorectal, breast, lung carcinoma, or osteosarcoma [120,121,122,123]. Unfortunately, the clinical trials have not been continued due to poor bioavailability of 2-ME [124,125]. Nonetheless, studies, including our group’s, are being performed to search for novel, better forms of drug formulation and/or derivatives of 2-ME [126,127,128]. 

2-ME is formed from 17β-estradiol (E2) by sequential hydroxylation and methylation of estrogens [118]. The first step is the oxidation at carbon 2 inside the aromatic A ring of estradiol which is catalyzed by the cytochrome P450 isoform 1A1 to give 2-hydroxyestradiol (2OHE2). Then, the hydroxyl group earlier added to 2OHE2 is swapped with a methyl group by a catechol-O-methyltransferase (COMT), which can be identified in numerous organs including liver, kidney, brain, mammary, and also in erythrocytes, to give a 2-ME molecule [118,129,130]. Importantly, COMT [118], which is widely distributed in the hippocampus where it catabolizes the catecholamine neurotransmitters, influences cognitive function, regulates dorsal hippocampal neurochemistry, and modulates hippocampus-dependent behaviours [131]. The level of E2 in hippocampus reaches even six times higher values than in plasma [132]. Thus, we suggest that 2-ME may be a metabolite of E2 also in brain structures [132].

2-ME achieves serum concentrations below 10 pg/mL in men, while in women from 18 to 63 pg/mL in the follicular phase of the menstrual cycle and from 31 to 138 pg/mL in the luteal phase. During pregnancy, the concentration of 2-ME in women may increase from 2035 to 10,691 pg/mL. After menopause, 2-ME concentrations in women range from 21 to 76 pg/mL [133,134]. In contrast, in pharmacological treatment even 1200 mg of 2-ME is used as a daily dose [120,121,122,123,135].

The molecular anticancer mechanism of action of 2-ME is not entirely understood yet, but it was already established that it generates ROS and RNS leading to nitro-oxidative stress inducing apoptosis [136,137,138]. The most important antiproliferative mechanisms include inhibition of microtubule dynamics, inhibition of neoangiogenesis, and regulation of extrinsic and intrinsic apoptotic pathways [118]. 2-ME interacts with tubulin and by their inhibition leads to cell growth inhibition and cytotoxic effect [139,140]. Moreover, it phosphorylates Bcl-2 and Bcl-xL, two members of the Bcl-2 family with antiapoptotic activity. Phosphorylation of these proteins reverse the antiapoptotic effects and occurs in several cell types due to activity of 2-ME [141,142]. In addition, it has been shown that 2-ME increases the level of BAX, reduces the concentration of Bcl-2, activates both Bak and BAX, and mitochondrial-dependent caspases [143]. 

Our own long-lasting studies revealed that 2-ME selectively induces and uncouples neuronal nitric oxide synthase (nNOS) in both cancer and neuronal cell lines, notably, at pharmacological and physiological concentrations [136,137,138,144]. From mechanistic point of view 2-ME increases the localization of nNOS in the cell nucleus, causing DNA damage from nitro-oxidative stress, which then causes cell cycle arrest and apoptosis in osteosarcoma cells [136,137,138,144,145,146]. The induction of nNOS and production of nitric oxide (NO) at physiological concentrations suggests the hypothesis that 2-ME in the human body is not only a metabolite of the active molecule, but also a self-acting hormone [137]. 

## 6. Activity of 2-ME in Neurons

It is also worth emphasizing that the above-mentioned mechanism of action of 2-ME is not only limited to the neoplastic cells themselves, but generally to all actively dividing cells, including neurons [147] as two sites of active neurogenesis remain in the adult brain—the dentate gyrus of the hippocampus and the subventricular part of the olfactory bulb [148]. Therefore, it is worth considering, whether 2-ME is used in anticancer therapy, it will have a toxic effect on brain cells. An interesting fact is that only pharmacological concentrations of 2-ME were proved to have a cytotoxic effect on HT22 cells [144]. However, the experimental conditions i.e., time of incubation has to be taken into consideration. On the other side, as evidenced by our group, 2-ME possesses genotoxic potential and selectively induces RNS production in hippocampal HT22 cell lines also at physiological relevant concentrations [144]. 2-ME selectively increases nNOS protein levels in a time-dependent manner [144]. Furthermore, the specific induction of nNOS by 2-ME seems to be unique for this molecule, as 2-ME did not affect endothelial and inducible nitric oxide synthase (eNOS, iNOS) levels [136]. Remarkably, 2-ME similarly increases nNOS protein levels in HT22 cells by constitutive enzyme expression [136]. 

Despite the fact that NO is not highly reactive and unstable, it can easily be oxidized to generate highly damaging reactive nitrogen species (RNS) like peroxynitrite or nitrogen dioxide [116,149,150,151]. A fingerprint of RNS, an indicator of nitro-oxidative stress under pathophysiological conditions, is 3-nitrotyrosine (3-NT) generated in the reaction of nitrating oxidants by protein tyrosine residues or free tyrosine [152,153,154,155]. Interestingly, augmented levels of nitrated proteins and 3-NT have been identified in numerous neurodegenerative diseases like including PD [156,157]. The elevated level of 3-NT turned out to coincide in neuronal and 2-ME-treated OS cells. Increased nNOS due to the action of 2-ME in OS cells is closely related to the elevated expression of 3-NT [136]. Indeed, we observed 2-ME-mediated increased level of 3-NT in both cancer and hippocampal cells [136]. By increasing the level of nNOS and 3-nitrotyrosine [136], 2_ME may under physiological and pharmacological conditions contribute to the development of neurodegenerative diseases by increasing the nitrated or nitrosylated forms of proteins [32]. What is more, α-syn activates nNOS in rat brain cells [158]. 

2-ME-mediated-induction of cell death was also performed on the SH-SY5Y neuroblastoma line—a childhood malignant tumor, resistant to pharmacotherapy [159,160]. Besides, SH-SY5Y cell line serves as an in vitro model of neurotoxicity due to its dopaminergic features [161]. When pharmacologically significant concentrations were used, 2-ME induced apoptosis in SH-SY5Y cells through NO production and decreased mitochondrial membrane potential [145].

Taking into consideration all above-mentioned data, some questions arise about 2-ME and its probable neurodegenerative features. As 2-ME is synthesized in the brain, and induces apoptosis in actively dividing cells, may this compound be toxic to neuronal cells? Could neurodegeneration be a side effect of chemotherapy? Can physiological concentrations of 2-ME protect the body against cancer development, but on the other hand, contribute to the development of a neurodegenerative disease, for instance PD?

The hypothesis is supported by the fact that the highest physiological concentrations of 2-ME are recorded in pregnant women and which, over 33% develop depression or memory loss [162]. The exact cause is still unknown, so the question is, is it related to the increased levels of 2-ME in the body? It is also worth considering the interesting “resistance” of PD patients to certain types of cancer [163]. Are levels of 2-ME elevated in their bodies and do they damage neurons, but protect them against cancer development? Additionally, are women more susceptible to neurodegeneration development as they have higher physiological concentrations of 2-ME in comparison to men? In addition, is 2-ME protecting females from cancerogenesis? Additionally, a considerable increase in the expression of COMT, an enzyme crucial of 2-ME production, was found in dopaminergic neurons of the PARK2-induced isogenic stem cell line which mimics the loss of PARK2 function [118,164]. Increased expression of COMT, exclusively in dopaminergic neurons of the substantia nigra, resulted in cataleptic behaviors related to diminished motor coordination in mice. A significant increase of COMT expression in dopamine neurons, especially in the substantia nigra, basically caused by PARK2 mutation, may result in the defect in synaptic dopamine transmission in the primary process of PD as a dramatic increase of COMT in patients carrying the PARK2 mutation were found [164]. Taking into consideration that COMT is widely distributed in the hippocampus [131], makes this region vulnerable to 2-ME mediated-nitro-oxidative stress damage [144]. On the other hand, Kanasaki et al. showed that pregnant mice deficient in COMT show a pre-eclampsia-like phenotype as a result of decrease of 2-ME, suggesting the potential use of 2-ME as a diagnostic marker for pre-eclampsia and as a curative drug [165].

## 7. Mitochondrial Abnormalities as a Mechanism of Neurodegeneration 

Mitochondria are organelles made of membranes, present in all eukaryotic cells, especially in tissues with high energy requirements, such as the brain and muscles. Due to their bacterial origin, they have their own genome and ability to autoreplicate. The major function of mitochondria is energy metabolism, mostly oxidative phosphorylation (OXPHOS) [4,166,167]. Beyond generation of adenosine triphosphate (ATP), mitochondria are the major cellular source of ROS, involved in calcium (Ca^2+^) homeostasis and in the regulation and induction of cell damaging pathways, which may constitute the basis of selective DA neurodegeneration in PD [4,166]. Moreover, mitochondrial oxidative stress is mediated by dopamine metabolism, which, when is in abundance outside of the synaptic vesicle in damaged neurons or, for instance, due to L-DOPA therapy, is metabolized by monoamine oxidase (MAO) or undertakes auto-oxidation and produces toxic ROS [168,169].

Cutting-edge research on mitochondrial bioenergetics, their dynamic interactions and their role in cellular homeostasis, have presented the neurodegenerative process of PD in a new light. Respiratory chain impairment is a key trait in sporadic PD patients and there is growing evidence that proteins encoded by genes associated with PD are linked to mitochondrial dysfunction [170]. PD-involved genes and their influence on mitochondrial functions are summarized in Table 2.

Interestingly, PD’s responsible protein, α-syn, is involved in many cell-devastating actions such as formation of pores on plasma membrane, deregulation of Ca^2+^ level, oxidative stress, causing lipid peroxidation and mitochondrial dysfunction in neurons [179,180]. α-syn is a presynaptic protein that is involved in the regulation of synaptic vesicle transport and in endocytosis [171]. α-syn interacts with mitochondria in substantia nigra inducing mitochondrial depolarization by inhibition of respiratory complex I at electron transport chain [172]. In addition, augmented cellular oxidative stress results in α-syn accumulation, which is a reason of mitochondrial dysfunction [171]. α-syn misfolding into oligomeric sheets and fibrillation causes mitochondrial damages [173]. Under normal physiological conditions, the interactions between mitochondria and the α-syn contributes to the health of the neurons, while the mechanisms for maintaining their homeostasis include proteasome pathways, autophagy and endo-lysosomal pathways. When imbalances occur, for example as a result of mitochondrial defects, the production of mitochondrial ROS increases, which promotes abnormal folding and aggregation of intracellular α-syn [181,182]. Moreover, α-syn participate in mitophagy and fission/fusion cycle [183].

## 8. Mitochondrial Biogenesis

The explanation of mitochondrial biogenesis is the growth and division of already-existing mitochondria [184]. This process is regulated by transcriptional activators like peroxisome proliferator-activated receptor gamma coactivator-1 alpha (PGC-1α), which cooperates with various transcription factors. What is more, high level of PGC-1α may lead to diagnosis of tumor dependency on mitochondrial mass as its expression indicates elevated mitochondrial respiration. However, in some cases the overexpression of PGC-1α may result in induction of apoptosis [185,186].

During an autopsy, decreased PGC-1α levels were found in brains of PD patients, in both the substantia nigra and in white blood cells [187]. Moreover, dopaminergic cells in PGC-1α knockout mice were more sensitive to 1-methyl-4-phenyl-1,2,3,6-tetrahydrodropyridine (MPTP), a well-known neurotoxin which causes selective degeneration of the substantia nigra, like in PD, after systemic administration [188,189,190]. However, PGC-1α overexpression reverses neurotoxicity induced by MPTP. Additionally, due to stimulation of PGC-1α by resveratrol, it exhibits neuroprotective properties [191]. Above-mentioned analyses indicate that PGC-1α plays a crucial role in PD pathophysiology, and may be a hopeful target for the therapy [192].

Recent studies prove that proteins with a post-translational modification by small ubiquitin-like modifier (SUMO) can disrupt with mitochondrial dynamics, which is crucial for neuronal function, and may play a fundamental role in PD pathogenesis. SUMOylation may reduce the amount of PARKIN available for mitochondrial recruitment and, by suppressing PGC-1α may reduce mitochondrial biogenesis. On other hand, mitochondrial fission can be controlled by dynamin related protein 1 (Drp-1) SUMOylation. A proper balance between the SUMOylation and the deSUMOylation of these proteins is necessary to guarantee adequate mitochondrial function in PD [193]. Moreover, Siddiqui et al. reported that reductions in PARKIN solubility and function, due to its oxidation, in a mouse model of age-related sporadic PD coincide with decreasedPGC-1α signaling. Furthermore, resumption of PGC-1α expression was found to abolish losses of mitochondrial function and degeneration of DA neurons in the substantia nigra pars compacta in the mouse-model [171]. The above findings suggest that the modulation od mitochondrial biogenesis is a promising therapeutic target for the treatment of PD.

## 9. Fusion/Fission

The alternative way to control the quality of mitochondria is a mitochondrial fusion/fission cycle. The cycle allows mitochondria to adapt to metabolic needs of the cell by isolation damaged mitochondria from the whole network. The mitochondrial fusion interconnects mitochondria to each other as a single continuous reticulum, whereas fission results in fragmented mitochondria of smaller dimensions. The process of fission is regulated by Drp1, while fusion of outer and inner mitochondrial membranes (OMM and IMM) is controlled by mitofusins MFN1 and MFN2 and optic-atrophy-1 (Opa1) [185,194,195,196]. 

Fission/fusion machinery is regulated by mitochondrial metabolism respiration and oxidative stress. Fission inhibition and fusion strengthening results in mitophagy reduction, thus strengthening of fission fuels mitophagy [194]. An imbalance between fission and fusion is often present in cancers, because increased fission activity and/or decreased fusion leads to fragmented mitochondrial network [185]. 

Growing evidence reveals that Drp-1 regulate mitochondrial fission, fusion, and mitophagy, to protect from neurodegeneration development in PD. Actually, not only Drp-1-mediated fission is essential for mitophagy that has a protective effect on neurons, but pathological mitochondrial fission and mitophagy either stimulate survival of neurons or lead to their death, suggesting that Drp-1 may play a key role in the pathogenesis of PD [197]. However, the biological effects Drp-1 inhibitors, such as Mdivi-1, still pose a question. Future research that investigates the mechanisms involved in Drp1 activity, may provide new therapeutic discoveries for treating neurodegeneration in PD [197]. 

Interestingly, pharmacological inhibition of Drp-1 by Mdivi-1 in PD rats treated with dopamine receptor D1 antagonist, significantly reversed behavioral deficits, adjusted mitochondrial functions, biogenesis and enhanced the number of newborn DAergic neurons in substantia nigra pars compacta. Inhibition of Drp-1 in rats with PD, resulting in increased levels of protein kinase-B/Akt and extracellular signal regulated kinase (ERK), induced neuroprotective effects. The above data suggest that the elimination of mitochondrial fission and enhanced neurogenesis of DAergic by the activity of dopamine D1 receptor may involve inhibition of Drp-1 resulting in improved behavioral features in PD rats. It is also noteworthy, that inhibition of Drp-1 by Mdivi-1 possibly returned these effects of D1 receptor agonist in D1 antagonist treated PD rats, indicating the involvement of Drp-1 in D1 receptor mediated signaling [198].

What is more, α-syn takes part in the fission/fusion cycle [183]. As Kamp et al. demonstrated, α- syn inhibits fusion of model membranes. The consequences of elevated α-syn levels on membrane fusion in vivo were investigated by life cell imaging in SH-SY5Y cells and *Caenorhabditis elegans.* α-syn induces mitochondrial fragmentation, whereas its downregulation leads to elongation of mitochondria [199]. The mechanism of action is still unknown, whereas the experiment supports the idea that α-syn attaches to the OMM and inhibits or reduces their fusion, because α-syn is not operating directly with proteins involved in fusion or fission pathways such as s Mfn1, Mfn2 and Opa1, Drp1 [199]. Later, Martinez et al. proved that overexpressed wild type α-syn results in moderated toxicity, ROS production and mitochondrial abnormalities in SH-SY5Y cells. In addition, α-syn induced the mitochondrial fragmentation in a Drp-1-dependent manner. However, elevated level of the fusion protein Opa-1 prevented both mitochondrial fission and cytotoxicity, suggesting α-syn independency from the protein [174]. 

## 10. Mitophagy

Mitophagy is the selective degradation of mitochondria, a type of autophagy, and is the primary mechanism for controlling the quality of mitochondria in cells. The role of mitophagy is to remove malfunctioning or damaged mitochondria, which is essential for proper cell physiology and tissue development [194]. Mitochondria-derived vesicles are directly degraded to lysosomes [200]. 

PD’s associated proteins—PINK1 and PARKIN—are crucial for accurate mitophagy initiation [175,176]. PD mutations of genes linked to PARKIN, PINK1 kinase and E3 ubiquitin ligase are a reason for disrupted mitochondrial homeostasis in PD [176]. PTEN-induced putative kinase 1 (PINK1, PARK6) and Parkin (PARK2) were for the first time classified as genetic factors of PD in which mitochondrial malfunctioning has been proposed as one of the reasons [177]. PINK1 accumulates on the OMM of damaged mitochondria, stimulates Parkin’s E3 ubiquitin ligase activity, and recruits Parkin to the mitochondrion. Then, Parkin ubiquitinates OMM proteins, such as voltage-dependent anion channel-1 (VDAC1), MFN 1/2 and Miro 1, resulting in their degradation by the proteasome to trigger selective autophagy [177,178]. 

## 11. Mitochondrial Biogenesis and Mitochondrial Dynamics as Targets for 2-ME

**Figure 1:**
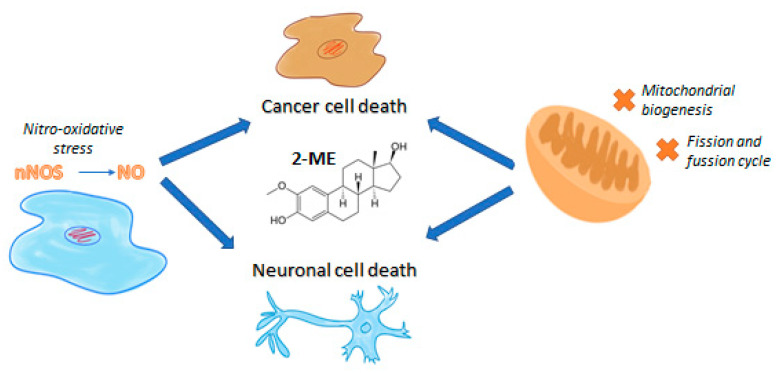
The anticancer mechanism of 2-ME and its plausible neurodegenerative features.

Cancer cells alter mitochondrial dynamics to regulate their bioenergetic and biosynthetic requirements to support tumor initiating and transformation abilities including proliferation, migration, and therapeutic resistance [201]. In our own studies it has been already demonstrated that 2-ME inhibits mitochondrial biogenesis especially at low physiological concentrations, targeting PGC-1α, COXI, and SIRT3 via nuclear recruitment of nNOS and NO generation [146]. What is more, 2-ME induces DRP-1 expression, leading to inhibition of mitochondrial dynamics and apoptosis of 143B OS cells [202]. The anticancer mechanism of 2-ME and its plausible neurodegenerative features are presented in Figure 1.

Using an osteosarcoma experimental model, we already proved that 2-ME inhibits mitochondrial biogenesis, specifically at low physiological concentrations, targeting PGC-1α, COXI, and SIRT3 due to the nuclear recruitment of nNOS. 2-ME was also evidenced to be a potent inhibitor of SIRT3 through binding to both the canonical inhibitor binding and allosteric sites. Moreover, mitochondrial biogenesis pathway regulation and SDHA were established for a first time as novel targets of 2-ME [146]. Interestingly, 2-ME was earlier demonstrated to affect mitochondrial biogenesis and mitochondria dynamics in OS 143B cells via its impact on microtubules [203]. 2-ME at both pharmacological and physiological doses increases mitochondrial fission and induces autophagy in cancer cells [204]. Subsequent, upregulated expression of Drp1 and BAX proteins by 2-ME strongly suggests the activation of the intrinsic apoptosis pathway. We further observed 2-ME-mediated regulation of glycolytic state of OS cells [204]. Previously in clinical trials, intravenous ATP has been shown to increase survival in patients with premature cancer. However, the activity of ATP via purinergic receptors may mediate tumor promoting activities in prostate and breast cancer cells [204,205]. Based on this discovery, we suggest that anticancer mechanism of 2-ME relies on selective nitro-oxidative stress generation controlling the mitochondrial dynamics, including inhibition of biogenesis and induction of mitochondrial fission, finally resulting in mitophagy and cancer cell death [202].

## 12. Conclusions

2-ME, a metabolite of E2, is exerting its own significant biological activity what may suggest that 2-ME, as its mother compound (E2), is a hormone per se. Certainly, 2-ME has effective anticancer properties mostly due to the nitro-oxidative stress induction as well as regulation of mitochondria dynamics and function in cancer cells. On the other hand, the activity of this compound, also at physiological level, may lead to neuronal cell death. This may propose the probable role in neurodegenerations development or a neurotoxic side effects of 2-ME in anticancer therapy. As hippocampal damage is crucial in neurodegenerations, understanding the influence of estrogens on hippocampal structure and function may put these devastating diseases in a new light and may result in novel, more efficient therapies.

Investigating the role of 2-ME in pathogenesis and progression of PD using in vivo models is currently under our investigation.

## Figures and Tables

**Table 1:** Shared pathogenetic factors for cancers and PD.

Mutated Genes and Pathogenetic Functions	Involvement in PD	Involvement in Cancer	Reference
α-synuclein	Crucial component of Lewy bodies	Accumulation and aggregation e.g., in melanoma, brain and glial tumors	[33,34,35,36,37]
Parkin	Loss of function; crucial for accurate mitophagy initiation	Loss of function; increased sensitiveness to some cancers; initiate a tumor formation process; mutations present on e.g., lung, liver, intestine, and brain cancers	[19,20,21,22,23,24,25]
PINK1	Loss of function; stabilize the mitochondrial membrane potential; deficiency impairs the plasticity of stratium and hippocampus	High expression in lung cancer; probable factor of chemo-resistance	[26,27,28,29]
Nitro-oxidative stress, mitochondrial dysfunction	Progression of neurodegeneration; damage DNA, lipid, and proteins; inducing apoptosis	Progression of cancer cells proliferation; damage DNA, lipid, and proteins; inducing apoptosis	[42,43,44,45]

**Table 2:** The summary of PD-involved genes and their influence on mitochondrial functions.

Mutated Gene	Description of Gene	Influence of Mutations on Mitochondrial Function	References
α-synuclein	Crucial component of Lewy bodies; regulate synaptic vesicle transportation and endocytosis	Disturbed mitochondrial trafficking; fragmented mitochondria; inhibition of respiratory complex I; misfolding into oligomeric which are toxic to the mitochondria; induces the mitochondrial fragmentation	[2,171,172,173,174]
PINK1	Kinase localized in mitochondria; crucial for accurate mitophagy initiation	Accumulates on the OMM of damaged mitochondria, and recruits Parkin to the dysfunctional mitochondrion	[175,176,177,178]
Parkin	Cytosolic E3 ubiquitin ligase located in mitochondria; crucial for accurate mitophagy initiation	Ubiquitinates outer mitochondrial membrane proteins and leads to their degradation by the proteasome	[175,176,177,178]
